# Cabozantinib for the Management of Metastatic Clear Cell Renal Cell Carcinoma

**DOI:** 10.15586/jkcvhl.2018.109

**Published:** 2018-10-08

**Authors:** Sharon J. Del Vecchio, Robert J. Ellis

**Affiliations:** 1 Centre for Kidney Disease Research, University of Queensland, Brisbane, Australia; 2 Translational Research Institute, Brisbane, Australia; 3 Department of Urology, Princess Alexandra Hospital, Brisbane, Australia

**Keywords:** cabozantinib, renal cell carcinoma, targeted therapy, tyrosine kinase inhibitor

## Abstract

Cabozantinib is a multi-tyrosine kinase inhibitor used for the treatment of various solid-organ tumours. It was recently approved as a first- and second-line therapeutic for the management of advanced/metastatic renal cell carcinoma based on the results of two randomised controlled trials. The phase III METEOR trial compared cabozantinib against everolimus as a second- or greater line therapy and found benefits in progression-free and overall survival, and the phase II CABOSUN trial compared cabozantinib against sunitinib as a first-line therapeutic and found benefits in terms of progression-free survival. This review briefly summarises how cabozantinib fits into current treatment paradigms for the management of advanced renal cell carcinoma.

## Introduction

The kidney is the 6th and 13th most common site of primary malignancy in men and women, respectively, in the developed world (1). Renal cell carcinoma (RCC) constitutes 90% of kidney cancers (2). Novel therapeutic agents for RCC have a high clinical utility, as a large number of patients (between 25 and 30%) are found to have metastatic disease at diagnosis (3). Treatment with targeted therapies has resulted in improved patient outcomes in the context of advanced metastatic RCC, with randomised controlled trials demonstrating improvements in both progression-free and overall survival compared with previous standard-of-care systemic therapies (4).

RCC represents a heterogeneous group of cancers that arise from the kidney. The most common histological variant of RCC is clear cell RCC (ccRCC), which comprises about 70% of RCC and has the highest metastatic potential. Other common subtypes include papillary RCC and chromophobe RCC; however, more than 15 histological subtypes have been described (5). The genetic basis of ccRCC is via a biallelic inactivation of the von Hippel-Lindau (VHL) tumour suppressor gene located on the short arm of chromosome 3 (3p25.3), which encodes for the degradation of hypoxia-inducible factor. This mutation leads to disruption of the oxygen-sensing pathway, thereby promoting angiogenesis and tumour proliferation/migration through the accumulation of vascular endothelial growth factor (VEGF), platelet-derived growth factor and fibroblast growth factor (6). Although VHL mutations are present in an estimated 70% of clear cell cancers, genetic heterogeneity is a hallmark of progressive disease with several other genes responsible for disease advancement and resistance to therapy (7, 8). Another oncogenic pathway implicated in ccRCC is the PI3K/Akt/mTOR cascade which is a known regulator of cellular metabolism and survival (9). Better understanding of the molecular signalling pathways implicated in RCC has driven the use and evolution of targeted therapeutics that have improved the standard of care for patients with advanced RCC. This review discusses the recently FDA-approved multi-kinase inhibitor, cabozantinib, and its role in the management of advanced RCC.

## History of Therapeutics in Advanced Renal Cell Carcinoma

Prior to the early 2000s, systemic therapies demonstrating survival benefit for patients with advanced RCC were limited to interleukin-2 (IL-2) and interferon alpha (IFN-α) that achieved a response in about 20% of patients and carried high toxicity profiles (10). Sorafenib, the first small-molecule oral multi-kinase inhibitor, gained FDA approval in December 2005 for the treatment of advanced RCC (11). The early-to-mid 2000s marked the beginning of the tyrosine kinase inhibitor (TKI) era for the treatment of advanced RCC, with TKIs appearing for the first time in European Association of Urology (EAU) recommendation guidelines for metastatic RCC therapy in 2006 (12). Large randomised clinical trials have since demonstrated the effectiveness of TKIs as a first-line therapy, with improved progression-free survival compared to treatment with interferon or placebo (13). Emerging resistance to TKIs has driven investigation into new molecular targets and the development of multi-kinase inhibitors.

## Mechanisms of Action of Cabozantinib

Cabozantinib is a multiple receptor tyrosine kinase inhibitor that initially gained FDA approval as a second-line therapy for patients with advanced RCC, who developed resistance to first-line agents, and has more recently gained approval as a first-line therapy. Targeting VEGF receptors 1–3, AXL, MET, RET, KIT, FLT3, ROS1, MER, TYRO3, TRKB and TIE-2, the mechanism of cabozantinib inhibits both the VEGF pathways, and downstream targets MET and AXL which are implicated in tumour resistance in patients treated with VEGF therapy alone, such as sunitinib (14, 15). Cabozantinib is predominantly metabolised by the liver and is a substrate of CYP3A4. Co-administration of strong inducers or inhibitors of CYP3A4 should be avoided in patients who are prescribed cabozantinib to avoid fluctuations in desired plasma concentration (16).

## Use of Cabozantinib for Treating Advanced Renal Cell Carcinoma

The first clinical use of cabozantinib in patients with RCC was in a phase I trial which included 25 patients who had failed standard systemic therapy (17). This trial demonstrated promising results, with 19 patients experiencing tumour regression. Cabozantinib was first approved by the FDA (Cabometyx™) as a second-line treatment for metastatic ccRCC in 2016 (18), following finalisation of the results of the phase III METEOR trial, which demonstrated benefits of cabozantinib over everolimus in terms of progression-free survival, overall survival and radiological tumour response in patients who had failed to respond to at least one tyrosine kinase inhibitor (Table 1) (19–21). Subgroup analyses indicated that efficacy in patients with skeletal metastases was particularly significant (22). The FDA subsequently approved cabozantinib as a first-line therapy for the management of metastatic ccRCC, following completion of the phase II CABOSUN trial, which demonstrated benefits of cabozantinib compared with sunitinib (which is the standard first-line therapy) in terms of progression-free survival and radiological tumour response (although there was no benefit in overall survival) (23, 24). A recently published case report demonstrated promising efficacy of cabozantinib in the management of ccRCC with brain metastases, which constitute <8% of all metastatic ccRCC but are classically associated with poor prognosis due to resistance to anticancer therapies (25).

**Table 1:** Clinical trials of cabozantinib in patients with metastatic ccRCC

Study	Population	Design	Findings
Phase I Trial (17)	25 patients with metastatic ccRCC who had failed standard systemic therapy (median of two prior systemic agents)	Phase I trial assessing safety and tolerability of 140 mg cabozantinib (PO daily) in patients with ccRCC	**Safety:** Adverse events were primary reason for discontinuation of drug for six patients, and dose reduction in 20 patients. **Efficacy:** Seven patients had a partial response, 13 patients had stable disease and 19 patients experienced tumour regression. Median progression-free survival was 12.9 months; median overall survival was 14.0 months.
Phase III Trial (METEOR) (19, 20, 21)	658 patients with metastatic ccRCC who had previously been treated with ≥1 VEGFR tyrosine kinase inhibitor and experienced disease progression within 6 months of most recent treatment	Phase III randomised trial (1:1 randomisation to each arm) assessing efficacy of 60 mg cabozantinib (PO daily; n = 330) compared with 10 mg everolimus (PO daily; n = 328)	**Primary endpoint:** *Progression-free survival—*better progression-free survival in cabozantinib arm compared with everolimus arm (HR: 0.51, 95% CI: 0.41–0.62). **Secondary endpoints:** *Overall survival—*better overall survival in cabozantinib arm compared with everolimus arm (HR: 0.66, 95% CI: 0.53–0.83). *Objective response per independent radiology review* – partial response in 17% (95% CI: 13–22%) of patients in the cabozantinib arm, compared with 3% (95% CI: 2–6%) in the everolimus arm. **Quality-of-life:** Similar responses to validated quality-of-life questionnaires between treatment arms; however, time to deterioration was longer in cabozantinib arm.
Phase II Trial (CABOSUN) (23, 24)	157 patients with metastatic ccRCC (with intermediate-poor prognosis) who had not previously received systemic treatment	Phase II randomised trial (1:1 randomisation to each arm) assessing efficacy of 60 mg cabozantinib (PO daily; n = 79) compared with 50 mg sunitinib (PO daily, 4 weeks on, 2 weeks off; n = 78)	**Primary endpoint:** *Progression-free survival—*better progression-free survival in cabozantinib arm compared with sunitinib arm (HR: 0.66, 95% CI: 0.46–0.95; updated analysis: HR: 0.48, 95% CI: 0.31–0.74). **Secondary endpoints:** *Overall survival—*better overall survival in the cabozantinib arm compared with sunitinib arm, although this did not reach pre-specified or conventional levels of statistical significance (HR: 0.80, 95% CI: 0.50–1.26). *Objective response per independent radiological review—*complete or partial response in 33% (95% CI: 23–44%) of patients in the cabozantinib arm compared with 12% (95% CI: 5.4–21%) in the sunitinib arm. **Safety:** Similar incidence and severity of adverse events across both treatment arms.

ccRCC, clear cell renal cell carcinoma; CI, confidence interval; HR, hazard ratio; PO, per oral.

Clinical guidelines produced by the EAU and the European Society for Medical Oncology currently recommend cabozantinib as a second-line therapy, following trial of combination therapy of ipilimumab plus nivolumab or standard tyrosine kinase inhibitor (26). EAU guidelines further state that, for patients with tumours of intermediate-poor prognosis (determined using the Heng score) (27), there is weak-level evidence for initiating cabozantinib as a first-line therapy in patients where use of ipilimumab plus nivolumab is contradicted or not feasible (Table 2) (26, 28).

**Table 2:** Clinical guidelines outlining the role of cabozantinib in the management of metastatic clear cell renal cell carcinoma

	Favourable prognosis	Intermediate-poor prognosis
European Association of Urology (26)	Offer cabozantinib as a second- or greater line therapy to patients who have been trialled on sunitinib, pazopanib or ipilimumab/nivolumab (strong evidence)	Offer cabozantinib to treatment-naïve patients when ipilimumab/nivolumab is not safe or feasible (weak evidence) Offer cabozantinib as a second- or greater line therapy for patients unresponsive to ipilimumab/nivolumab (strong evidence)
European Society for Medical Oncology (28)	Offer cabozantinib to all patients as a second- or greater line therapy following initial trial of any tyrosine kinase inhibitor (strong evidence)	

## Safety Profile and Side Effects

In both the METEOR and CABOSUN trials, there was an equivalent safety profile of cabozantinib and both everolimus and sunitinib, with daily oral doses of cabozantinib of 60 mg (20, 23). Adverse events associated with cabozantinib included hypertension, palmar-plantar erythrodyesthesia syndrome, diarrhoea, nausea/vomiting, anorexia/decreased appetite, fatigue and stomatitis/oral mucositis (29, 30). Prophylactic and supportive management should be initiated to reduce symptom burden (29). Eating should be avoided 2 h before and 1 h after taking cabozantinib (31). If cabozantinib is poorly tolerated despite prophylactic and supportive management of side effects, stepwise dose reduction in 20 mg increments is recommended (29).

## Cost-effectiveness

A recent systematic review which evaluated the cost-effectiveness of cabozantinib compared with other second-line therapies found that although cabozantinib was associated with favourable progression-free and overall survival, it was also one of the most expensive drugs (compared with everolimus, axitinib, nivolumab and sunitinib). In US dollars, costs of cabozantinib per patient per month of progression-free and overall survival were $17,864 and $11,166–$12,303, compared with $16,889 and $8,569–$9,724 with everolimus (32).

## Conclusion

There is good evidence for the use of cabozantinib as a second-line therapy for the management of advanced RCC, and it may provide an alternative to ipilimumab plus nivolumab as a first-line agent in patients with intermediate-poor prognosis. Although associated with marginally higher costs, randomised controlled trials of cabozantinib compared with standard therapeutics demonstrate evidence of improved progression-free and overall survival.

## Conflict of Interest

The authors declare no potential conflicts of interest with respect to research, authorship and/or publication of this article.
